# Sonographic evaluation of the immediate effects of eccentric heel drop exercise on Achilles tendon and gastrocnemius muscle stiffness using shear wave elastography

**DOI:** 10.7717/peerj.3592

**Published:** 2017-07-19

**Authors:** Wilson K.C. Leung, KL Chu, Christopher Lai

**Affiliations:** Department of Health Technology and Informatics, Hong Kong Polytechnic University, Hung Hom, Hong Kong

**Keywords:** Heel drop exercise, Achilles tendon, Gastrocnemius muscle, Shear wave elastography, Mechanical adaptations

## Abstract

**Background:**

Mechanical loading is crucial for muscle and tendon tissue remodeling. Eccentric heel drop exercise has been proven to be effective in the management of Achilles tendinopathy, yet its induced change in the mechanical property (i.e., stiffness) of the Achilles tendon (AT), medial and lateral gastrocnemius muscles (MG and LG) was unknown. Given that shear wave elastography has emerged as a powerful tool in assessing soft tissue stiffness with promising intra- and inter-operator reliability, the objective of this study was hence to characterize the stiffness of the AT, MG and LG in response to an acute bout of eccentric heel drop exercise.

**Methods:**

Forty-five healthy young adults (36 males and nine females) performed 10 sets of 15-repetition heel drop exercise on their dominant leg with fully-extended knee, during which the AT and gastrocnemius muscles, but not soleus, were highly stretched. Before and immediately after the heel drop exercise, elastic moduli of the AT, MG and LG were measured by shear wave elastography.

**Results:**

After the heel drop exercise, the stiffness of AT increased significantly by 41.8 + 33.5% (*P* < 0.001), whereas the increases in the MG and LG stiffness were found to be more drastic by 75 + 47.7% (*P* < 0.001) and 71.7 + 51.8% (*P* < 0.001), respectively. Regarding the AT, MG and LG stiffness measurements, the inter-operator reliability was 0.940, 0.987 and 0.986, and the intra-operator reliability was 0.916 to 0.978, 0.801 to 0.961 and 0.889 to 0.985, respectively.

**Discussion:**

The gastrocnemius muscles were shown to bear larger mechanical loads than the AT during an acute bout of eccentric heel drop exercise. The findings from this pilot study shed some light on how and to what extent the AT and gastrocnemius muscles mechanically responds to an isolated set of heel drop exercise. Taken together, appropriate eccentric load might potentially benefit mechanical adaptations of the AT and gastrocnemius muscles in the rehabilitation of patients with Achilles tendinopathy.

## Introduction

The calf muscle situated at the posterior side of the lower legs is composed of gastrocnemius (medial (MG) and lateral (LG)) and soleus, in connection with Achilles tendon (AT) to form a musculotendinous unit. The muscles contract concentrically to plantar flex the ankle, leading to an action-reaction force couple against the ground for locomotion. The AT is the largest and strongest tendon, which is responsible for withstanding tension and transmitting muscular force during locomotion, and there is a linear force-length relationship of the AT (Tardioli, Malliaras & Maffulli, 2012). Since abnormal, acute and subacute tendon adaptation responses immediately following weight-bearing exercises could largely contribute to tendinopathy (Tardioli, Malliaras & Maffulli, 2012), it is imperative to determine appropriate mechanical loading of each type of exercise training program as reflected by immediate changes in tendon stiffness.

Heel drop exercise is a type of eccentric exercise, which has been empirically proven to be an effective, nonsurgical regimen for Achilles tendinopathy (Alfredson et al., 1998; Maffulli et al., 2008; Sayana & Maffulli, 2007; Van der Plas et al., 2012). Eccentric calf muscle exercise was demonstrated to induce acute changes on the transverse morphology and strain of the AT in healthy, recreationally active adults (Grigg, Wearing & Smeathers, 2009; Obst, Newsham-West & Barrett, 2015), and thickness alteration of the AT assessed by ultrasonography was conferred as a promising prognostic parameter for predicting Achilles tendinopathy within six to 12 months in asymptomatic and symptomatic patients (Bakkegaard et al., 2015; Fredberg & Bolvig, 2002). Eccentric heel drops involve maximal ankle dorsiflexion, eccentrically lengthened calf muscles and reduced ankle planter flexion moments (Alfredson et al., 1998; Maffulli et al., 2008; Jeong et al., 2014; Sayana & Maffulli, 2007). A 5-year, within-subject, prospective study in patients with chronic midportion Achilles tendinopathy revealed Alfredson’s heel drops (i.e., 180 repetitions per day Alfredson et al., 1998) significantly improve validated Victorian Institute of Sports Assessment-Achilles questionnaire (VISA-A) score (Van der Plas et al., 2012), which primarily measures pain, functions in daily activities and physical activity of the AT (Robinson et al., 2001). It is known that modification of the strain ratio on muscles to tendons could change the relative stretch of the tendon and hence reduces the injury risks to the tendon with joint motion (Maffulli, Sharma & Luscombe, 2004). Although there was a study showing a 6-week eccentric heel drop program could improve plantar flexor muscle-tendon tissue characteristics in terms of dorsiflexion range of motion and passive resistive torque (Mahieu et al., 2008), there is still no empirical study revealing the underlying mechanism(s) associated with the mechanical interplay of each muscle and tendon of the calf in response to heel drop exercise.

With real-time, dynamic properties and quantitative capability in measuring soft tissue stiffness, shear wave elastography (SWE) has emerged as a powerful tool for accurately measuring muscle hardness (Eby et al., 2013) and tendon stiffness (Chen et al., 2013; Chiu et al., 2016; Siu et al., 2016). Comparing with conventional strain elastography, SWE is technically less operator-dependent as it largely relies on controlled ultrasound push beams rather than manually on tissue compression by sonographers for tissue deformation (Inami & Kawakami, 2016). It has also been proven to possess high intra-operator (ranging from 0.751 to 0.941) and moderate inter-operator reliability (0.585 and 0.749) in assessing the AT stiffness in both Chinese athletes and non-athletes, respectively (Chiu et al., 2016; Siu et al., 2016). Considering the fact that eccentric heel drop exercise with extended (i.e., straightened), instead of flexed, knee could confer larger changes in the triceps surae length, especially gastrocnemius muscles, and hence a greater amount of AT loads (Weinert-Aplin, Bull & McGregor, 2015), the objective of this study was thus to investigate the immediate effects of a heel drop exercise with full knee extension on the stiffness of AT, MG and LG using SWE. Given short-term, repeated eccentric loading of the calf muscle could confer stiffer muscle and tendon properties (O’Neill, Watson & Barry, 2015), we hypothesized that an acute bout of eccentric heel drop exercise with fully-extended knee for 10 × 15 repetitions could increase the stiffness of AT, MG and LG.

## Materials and Methods

### Study design

This interventional study adopted within-subject repeated measures design. A convenience sample of Hong Kong Chinese healthy subjects aged between 18 and 25 that identified by face-to-face Physical Activity Readiness Questionnaire (National Academy of Sports Medicine, 2014) were drawn from a local university. All sonographic data were collected by trained sonographers within one experimental session. The reporting of this manuscript aligned with the guidelines of Template for Intervention Description and Replication (Hoffmann et al., 2014). This study has been approved by the Human Subjects Ethics Subcommittee of the Hong Kong Polytechnic University (HSEARS20160914002).

### Participants

Forty-five healthy college students (36 males and nine females; 21.2 ± 1.3 years old) were voluntarily recruited. Subjects without ankylosing spondylitis, rheumatoid arthritis, gout, past AT injuries, and a history of corticosteroid injections were included. Neither of them was a competitive athlete nor receiving any regular resistance training. Before the commencement of this study, all participants were fully informed about the experimental procedures, and written informed consents were obtained.

### Heel drop exercise protocol

Each subject completed a total of 150 heel drops (15 repetitions  × 10 sets, 30-second rest after each set), with a frequency of 2 s per repetition (0.5 Hz). The heel drops of our study followed the exercise regimen as suggested by previous studies (Alfredson et al., 1998; Maffulli et al., 2008; Jeong et al., 2014; Sayana & Maffulli, 2007). The subjects began with shifting their body mass on their forefoot of their dominant legs at slight ankle plantar flexion, during which the gastrocnemius muscles had been loaded slowly until maximum dorsiflexion was reached. In order to return to baseline, the body mass was therefore shifted to the non-dominant leg to raise the body. The knee of the dominant leg during eccentric phase was kept at full extension to maintain greater gastrocnemius activation (Maffulli et al., 2008; Weinert-Aplin, Bull & McGregor, 2015). A trainer supervised the heel drop trials, and ensured that each participant correctly performed the heel drop as instructed.

### Reliability tests

Six operators first performed the intra-rater reliability test on AT, MG and LG stiffness in five subjects. All measurements were repeated by three times in the same scanning session. Two operators with the highest intra-rater reliability were selected and conducted the inter-rater reliability test on another five subjects, and the one with the highest intra-rater reliability was selected to perform the AT, MG and LG stiffness measurements in the main study.

### Sonographic examination of the AT, MG and LG stiffness

**Figure 1:**
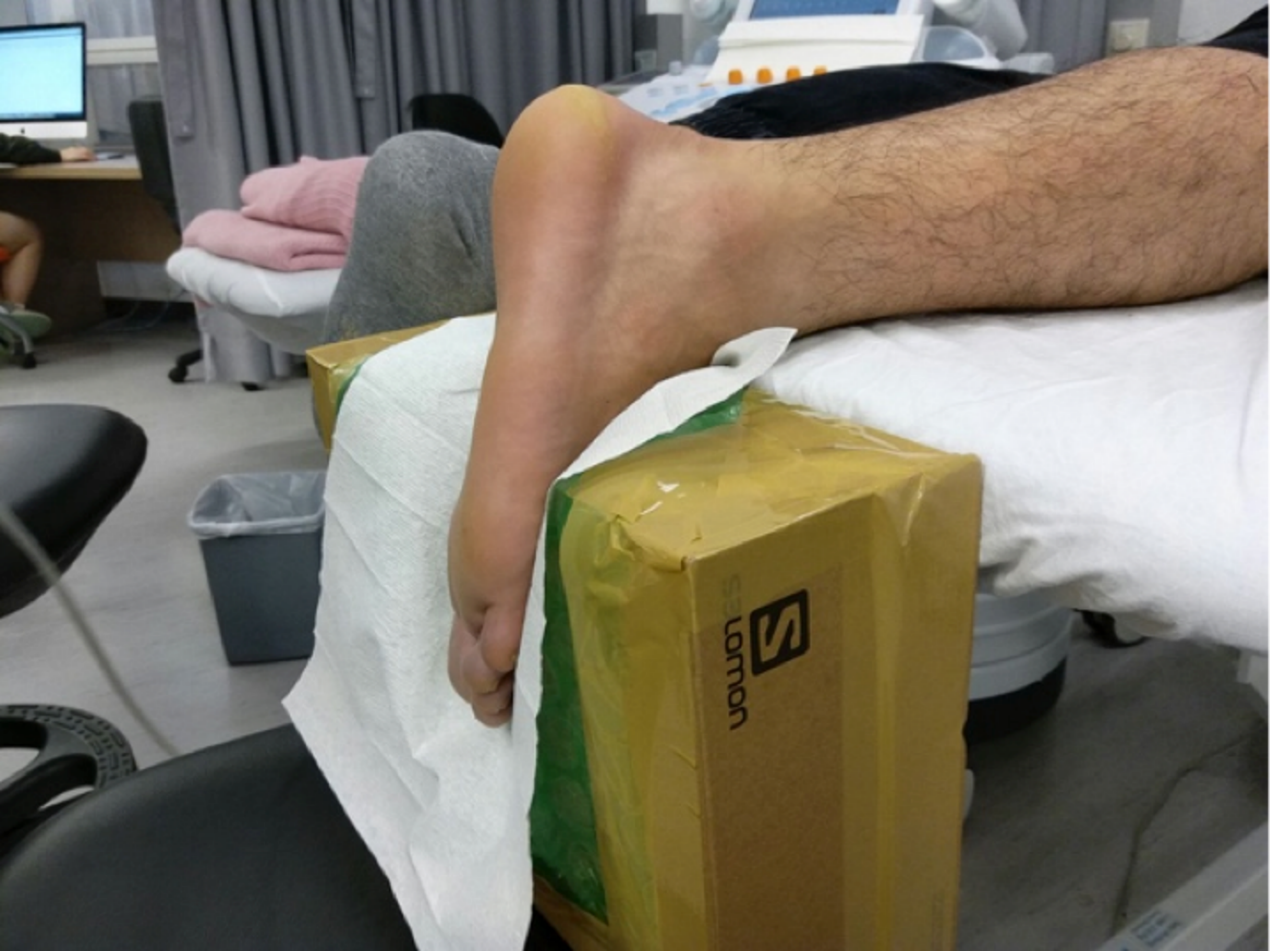
The set-up for examining the stiffness of the Achilles tendon and gastrocnemius muscles by shear wave elastography. The subject’s ankle was fixed by an in-house inclined board at 30°plantar flexion without ankle inversion and eversion.

As previously described, the passive torque at 30°ankle plantar flexion was approaching zero, and was considered as the most appropriate posture to reflect the slack lengths and stiffness of the AT and the gastrocnemius muscles (Akagi & Takahashi, 2013; Kawakami, Ichinose & Fukunaga, 1998; Riener & Edrich, 1999). An in-house, specially designed inclined board was made to fix the ankle of the subjects at 30°plantar flexion without ankle inversion or eversion (Fig. 1). The SWE examinations were carried out using an Aixplorer ultrasound unit (Supersonic Imaging, Aix-en-Provence, France) in conjunction with the Supersonic Super Linear™ SL15-4 Probe with a linear array of 4–15 MHz bandwidth.

**Figure 2:**
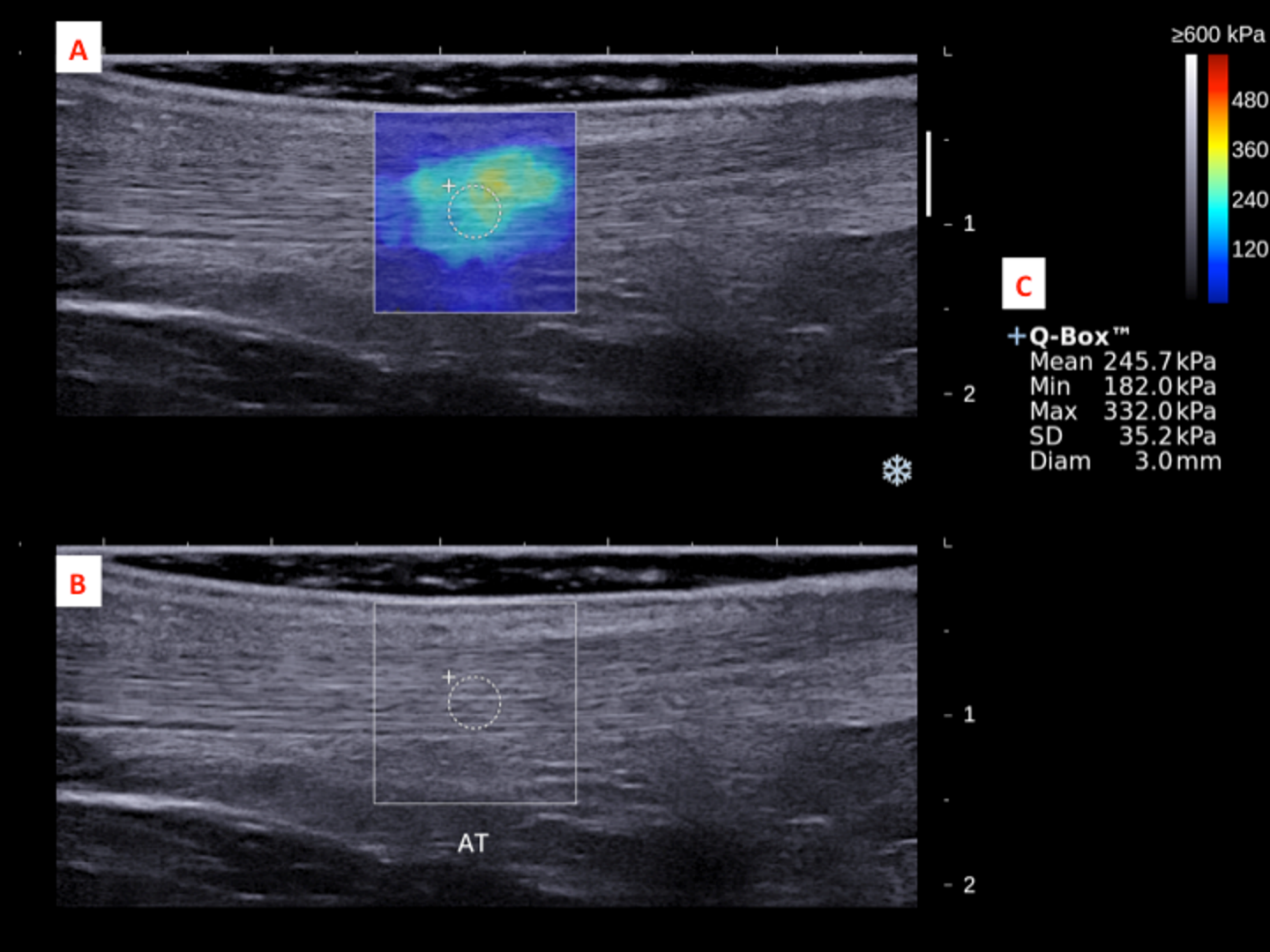
A shear wave elastogram (A) and the corresponding grey-scale ultra-sonogram (B) in the longitudinal axis of an Achilles tendon (AT) on the dominant leg of a subject after a heel drop exercise. (A) Q-box with a diameter of 3-mm was placed in the corresponding B-mode image (B) along the longitudinal axis of the AT for the tendon stiffness assessment. Different stiffness parameters were shown in (C).

**Figure 3:**
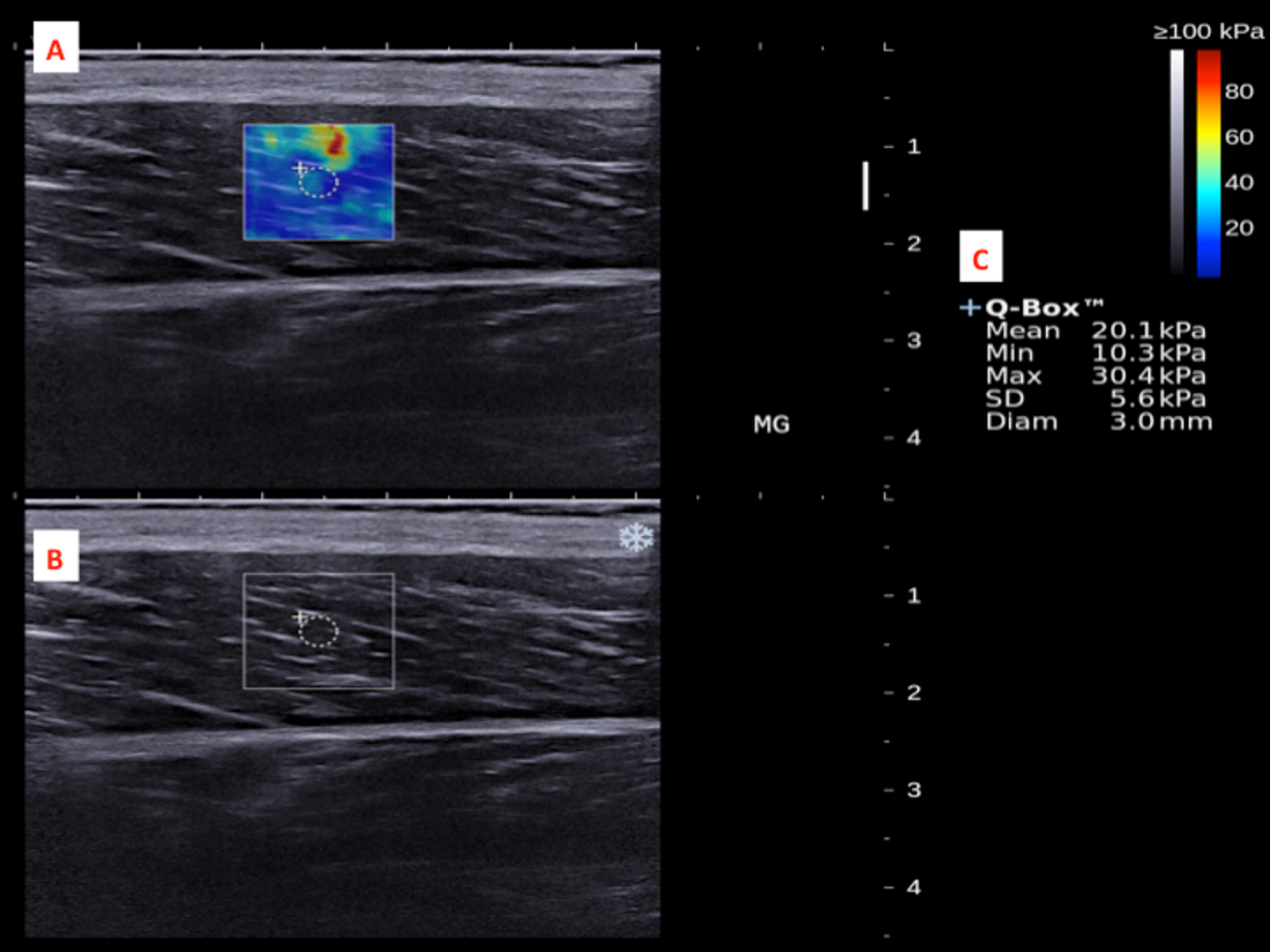
A shear wave elastogram (A) and the corresponding grey-scale ultra-sonogram (B) in the longitudinal axis of a medial gastrocnemius muscle (MG) on the dominant leg of a subject after a heel drop exercise. (A) Q-box with a diameter of 3-mm was placed in the corresponding B-mode image (B) along the longitudinal axis of the MG for the muscle stiffness assessment. Different stiffness parameters were shown in (C).

**Figure 4:**
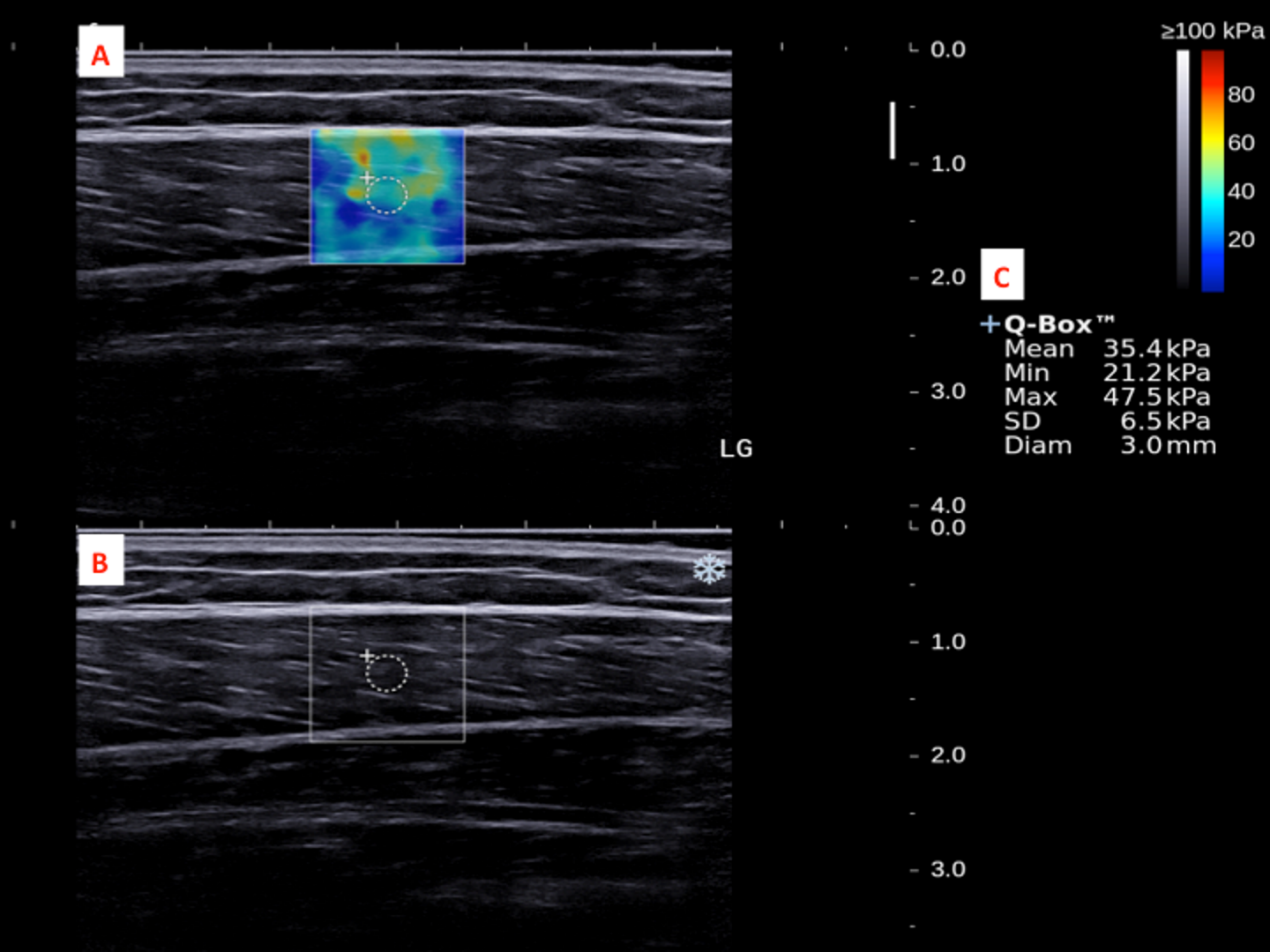
A shear wave elastogram (A) and the corresponding grey-scale ultra-sonogram (B) in the longitudinal axis of a lateral gastrocnemius muscle (LG) on the dominant leg of a subject after a heel drop exercise. (A) Q-box with a diameter of 3-mm was placed in the corresponding B-mode image (B) along the longitudinal axis of the LG for the muscle stiffness assessment. Different stiffness parameters were shown in (C).

The dominant legs of all the subjects were determined using a set of questions such as kicking a football, stepping on a bug, writing a word with a foot and taking a step forward, and the leg that they preferred the most (Velotta et al., 2011). All subjects lied prone on the examination couch and rested their dominant leg onto the inclined board with a piece of tissue on top for hygiene. The position of the subjects was adjusted so that the dorsum of their feet was in contact with the inclined board at 30° plantarflexion. The SWE measurements were made at the standardized level of the medial malleolus for the AT (Chiu et al., 2016; Siu et al., 2016) and at 30% leg length from femoral condyles for gastrocnemius muscles according to the ultrasound tape method (Barber, Barrett & Lichtwark, 2011). All the measurement sites were marked for standardization. Thick transmission gel was used to help optimize SWE measurement and image visualization. Images at the optimal position were captured, and a 3-mm diameter Q-box was placed at the region of interest for the measurement of elastic modulus in kPa (Figs. 2– 4). Three measurements were made and the average was recorded for data analyses.

### Statistical analyses

Intra-class correlation coefficient models 2 and 3 were used to assess the reliability of AT, MG and LG stiffness measurements among and within the operators, respectively. All data were presented as mean ± SD (standard deviation), and tested for normality using the Shapiro–Wilk test (Tables S1–S3). Only a dataset with *P* > 0.05 was considered as parametrically distributed. If one of the datasets for comparison was in nonparametric distribution, nonparametric tests would be performed. Either Paired *t*-test or Wilcoxon signed-rank test was used to examine the stiffness difference of the AT, MG and LG muscles before and immediately after the heel drops (Table 1). The Friedman test, which is a nonparametric alternative to the one-way analysis of variance (ANOVA), was used to detect differences in the percentage stiffness changes across the AT, MG and LG after the heel drop exercise. Since then, the group differences were evaluated by Mann–Whitney *U* test (Table 2). The level of significance was set to α = 0.05 given a two-tailed test had been considered. All statistical analyses were performed using Statistical Package for the Social Sciences (IBM SPSS, Version 24). Raw datasets were presented in Tables S1–S3.

**Table 1:** Achilles tendon and gastrocnemius muscle stiffness before and after the heel drop exercise (*n* = 45).

	Pre-		Post-		*P*-value
Stiffness (kPa)	Mean	SD	Mean	SD	
AT	254.6	59.8	346.1	58.2	<0.001
MG	16.9	3.8	28.4	5.1	<0.001
LG	16.0	4.0	26.3	6.0	<0.001

**Notes.**

AT Achilles tendon MG medial gastrocnemius LG lateral gastrocnemius SD standard deviation

**Table 2:** Percentage changes in stiffness of Achilles tendon and gastrocnemius muscle after the heel drop exercise (*n* = 45).

	% stiffness change		*P*-value
	Mean	SD	
AT	41.8	33.5	AT:MG, <0.001
MG	75.0	47.7	AT:LG, 0.009
LG	71.7	51.8	MG:LG, 0.62

**Notes.**

AT Achilles tendon MG medial gastrocnemius LG lateral gastrocnemius SD standard deviation

## Results

For the AT, MG, and LG stiffness measurements, the inter-operator reliability was 0.940, 0.987, and 0.986, and the intra-operator reliability ranged from 0.916 to 0.978, from 0.801 to 0.961, and from 0.889 to 0.985, respectively.

Two outliers defined as ≥1.5 times of the interquartile range away from either the first or third quartiles were identified in the datasets of the pre- and pos *t*-test MG muscle stiffness (32.7 and 44.3 kPa, respectively), and therefore the cases were excluded for pairwise comparisons. There were significant increases in the stiffness of the AT (from 254.6 ± 59.8 to 346.1 ± 58.2 kPa, *P* < 0.001), MG (from 16.9 ±3.8 to 28.4 ± 5.1 kPa, *P* < 0.001) and LG (from 16.7 ± 3.4 to 28.9 ± 5.7 kPa, *P* < 0.001) after the heel drop exercise (Table 1).

The Friedman test revealed a significant difference in the percentage changes of the stiffness across the AT (41.8 ± 33.5%), MG (75.0 ± 47.7%) and LG (71.7 ± 51.8%) (*Q* = 14.8, *P* < 0.001). It was shown the percentage changes in the stiffness of the MG and LG after the heel drops were greater than that of the AT (*P* < 0.001 and *P* = 0.009, respectively) (Table 2), whereas there was no significant difference in the percentage changes of the stiffness between the two gastrocnemius muscles (*P* = 0.62).

## Discussion

Pains and disorders of the AT are common in competitive and recreational athletes, and sedentary population (Claessen et al., 2014). Alfredson et al. (1998) first empirically reported the benefits of a short-term eccentric heel drop training (i.e., 12-week) in the rehabilitation of patients who had long been diagnosed with Achilles tendinopathy. The long-term outcomes of patients with midportion Achilles tendionopathy treated with the Alfredson’s heel drops (i.e., 5-year) measured by VISA-A questionnaire were also substantiated (Van der Plas et al., 2012). To understand the effects of a training program, it is necessary to first examine the immediate effects of an isolated set of specific exercise modes and doses on the participants. The present study is the first original research to report stiffer AT and gastrocnemius muscles after an acute bout of eccentric heel drop exercise in healthy young adults. Comparing with Chiu et al. (2016)’s (inter-operator reliability = 0.749, intra-operator reliability = 0.751–0.941) and Siu et al. (2016)’s (inter-operator reliability = 0.585, intra-operator reliability = 0.803–0.845) studies, we obtained much higher inter-operator (0.940) and intra-operator (0.916–0.978)reliability of the SWE measurement on the AT stiffness possibly due to the standardized level at medial malleolus for the measurements. As suggested by Peltz et al. (2013), the measurement repeatability is largely dependent on the relative position of transducer on tendon. We also achieved remarkably high intra-operator (MG = 0.801–0.961, LG = 0.889–0.985) and inter-operator (MG = 0.987, LG = 0.986) reliability of the MG and LG stiffness measurements versus Cortez et al. (2016)’s SWE measurements of the gastrocnemius medialis in healthy adults with intra-operator (0.690–0.700)and inter-operator (0.730) reliability.

However, eccentric training program for 6 weeks was shown to have no significant effect on the AT stiffness of recreational athletes (Mahieu et al., 2008). There are several reasons for the inconsistency of our findings. First, our eccentric heel drop protocol (10 ×15 repetitions) was not comparable to Mahieu et al. (2008)’s protocol (3 ×15 repetitions). Based on compatibility with modern busy lifestyle (i.e., little leisure time), our protocol (150 repetitions in total) was modified from the classical Alfredson’s heel drops (180 repetitions in total) (Alfredson et al., 1998), which is time consuming (Van der Plas et al., 2012). Second, the study by Mahieu et al. (2008) mentioned they adopted classical Alfredson’s heel drops, which actually include two types of eccentric exercises with knee straight and bent (Alfredson et al., 1998). In the present study, the participants only performed extended-knee mode of eccentric heel drop. As previously described, eccentric heel drop exercise with extended (i.e., straightened), instead of flexed, knee could confer a larger eccentric load of the AT (Weinert-Aplin, Bull & McGregor, 2015). Third, in Mahieu et al. (2008)’s study, the left legs or ankles of their participants were evaluated, whereas we were measuring the changes of the AT on the dominant legs (right-leg dominant (*n* = 42), left-leg dominant (*n* = 3)) in response to a heel drop exercise. In fact, there are distinct loading profiles between both legs conferring asymmetric tendon properties (Bohm et al., 2015). Someone define the ball-kicking side (i.e., the leg of which we prefer most) as the dominant leg based on higher AT stiffness (Bohm et al., 2015; Siu et al., 2016), whereas higher prevalence of tendon rupture signifies dominant tendency of another leg (Chiu et al., 2016; Pang & Ying, 2006). As suggested, there are different extents of the AT stiffness changes between both legs in response to immediate weight-bearing loads, and significant changes only occur in the leg of which we prefer most (Chiu et al., 2016). Forth, Mahieu et al. investigated the immediate effects of the heel drop exercise in recreational athletes, whereas we examined the effects in healthy college students, whom the type, frequency and duration of leisure-time physical activity were not considered (Booth, 2000). As suggested, higher thickness, cross-sectional area and stiffness of the AT were evident in frequent exercisers (having ≥6-h weight-bearing exercise per week) than age-matched infrequent exercisers (Siu et al., 2016; Ying et al., 2003). Also, there was a low prevalence of asymptomatic Achilles tendinopathy in active young adults (Joseph et al., 2012), implicating a prominent effect of physical activity on the AT properties. Despite immediate, short-term responses (biochemical, biomechanical and morphological) of the AT to different types of single exercise (Tardioli, Malliaras & Maffulli, 2012), there is still a lack of direct evidence linking acute changes of the AT after a single bout of exercise to the observed long-term adaptations with training (Obst, Barrett & Newsham-West, 2013). In order to tailor appropriate mechanical loads of a training on each individual, it is important to evaluate the immediate effects of each isolated set of specific exercise modes and prescriptions on the AT properties.

In a systematic review of peer-reviewed articles using a wide range of electronic databases on the subject matter of “immediate and short-term effects of exercise on tendon structure”, most relevant studies revealed a reduction in the AT stiffness after exercise (Tardioli, Malliaras & Maffulli, 2012). Specifically, there was a significant reduction in the AT stiffness immediately following 6 × 8-sec maximal voluntary isometric contractions in healthy subjects (Kay & Blazevich, 2009). The inconsistency of our findings could be explained by the fact that the eccentric heel drop exercise is in a different mode of contraction to isometric exercise. Another study also showed static stretching significantly decreased the AT stiffness on the right ankle in recreationally-active male subjects (Kubo et al., 2001), yet whether the ankle is on dominant side was not considered. As aforementioned, dominant and non-dominant ankles could have differential mechanical responses to immediate weight-bearing loads (Chiu et al., 2016). Most importantly, physical activity of the participants should not be ignored (Booth, 2000; Joseph et al., 2012; Siu et al., 2016; Ying et al., 2003).

In the present study, the extent of change in stiffness in the gastrocnemius muscles and AT during a heel drop exercise were uneven; MG and LG were shown to bear larger immediate eccentric load than AT. In fact, altered strain ratios of muscles to tendons could reduce the risk of stress on tendons rising to a level that might cause injury during normal motion, given the strain exerted on the tendon remains at a level of <4% (Maffulli, Sharma & Luscombe, 2004). Besides, restoring muscle and tendon elasticity by stretching and strengthening of the triceps surae muscles and AT is crucial for preserving functional ability of the musculotendinous unit, improving plantar flexor extensibility, and reducing strain on the AT during joint motion (Maffulli, Sharma & Luscombe, 2004). Superior to static stretching which was associated with impaired muscle performance (Kay & Blazevich, 2012; Simic, Sarabon & Markovic, 2013), eccentric heel drop training (similar to static stretching) was shown to be effective in relieving symptoms of Achilles tendinopathy without any reported adverse impact (Alfredson et al., 1998; Maffulli, Sharma & Luscombe, 2004; Maffulli et al., 2008; Sayana & Maffulli, 2007; Van der Plas et al., 2012). Thus, further investigations on various prescriptions of eccentric heel drop should be deserved to further pinpoint the ideal eccentric load in the mechanical adaptation of the calf muscle and tendon tissues.

There are several limitations needed to be addressed. First, as aforesaid, the physical activity of the participants was not considered as one of the eligibility criteria in our study as physical activity, especially leisure-time or weight-bearing exercises, could considerably influence the mechanical and morphological properties, and overall health of the AT (Booth, 2000; Joseph et al., 2012; Siu et al., 2016; Ying et al., 2003). Second, since Achilles tendinopathy is etiologically multifactorial, and complex in nature (Claessen et al., 2014), the subjects should be matched with or exclusion criteria should include all possible conditions related to the tendinopathy, for example, obesity (Franceschi et al., 2014), myofascial pain syndrome (Calvo-Lobo et al., 2017), hallux valgus (Lobo et al., 2016) and pes planus (Angin et al., 2014), special medications such as long-term statin and aromatase inhibitors consumptions (De Oliveira et al., 2015; Kirchgesner et al., 2014), and distorted redox status (Bestwick & Maffulli, 2004). Third, a randomized, controlled experimental setting in a larger sample of the trained and untrained should be considered. Fourth, the sample size of all outcome measures in the present study were not estimated by a power analysis, but the size of the experimental group (*n* = 45) was similar to or even much larger than that in other similar studies (Jeong et al., 2014; Mahieu et al., 2008; Obst, Newsham-West & Barrett, 2015; Weinert-Aplin, Bull & McGregor, 2015). Last but not least, the gender issue in tendinopathy should have further study under a better structured experimental design since the presence of female dominance predisposing to soft tissue injury during physical activity has been recently provoked (Astill et al., 2017). However, we did not observe any significant gender disparity in the percentage changes of AT (male = 46.0 ± 35.3%, female = 25.0 ±18.1%; *P* = 0.13), MG (male = 76.5 ± 51.6%, female = 68.6 ± 28.1%; *P* = 0.94), and LG stiffness (male = 66.4 ± 48.2%, female = 92.6 ± 63.1%; *P* = 0.20) immediately after a heel drop exercise in our study.

## Conclusions

In summary, it is shown that an acute bout of eccentric heel drop exercise results in stiffer AT and gastrocnemius muscles; gastrocnemius muscles might bear larger mechanical loads than the AT during an eccentric heel drop motion. Completion of this study could serve as a milestone to propose further study examining these variables in a larger sample size and even special populations with clinical tendon conditions.

##  Supplemental Information

10.7717/peerj.3592/supp-1 Data S1 Raw data

## Additional Information and Declarations

### Data Availability

The following information was supplied regarding data availability: The raw data has been provided as Data S1.
