# Supplementary material for: Sonographic evaluation of the immediate effects of eccentric heel drop exercise on Achilles tendon and gastrocnemius muscle stiffness using shear wave elastography
Source: PeerJ. 2017 Jul 19;5:e3592. doi: 10.7717/peerj.3592 (PMC5520961; doi:10.7717/peerj.3592)
Supplement: Data S1 [file peerj-05-3592-s001.docx]

**Supplemental Information**

**Table S1.** The average elastic modulus, in kPa, of the **Achilles tendons** before and immediately after a heel drop exercise (n=45) (raw data).

| **Subjects** | **Sex** | **Pre-** | **Post-** | **% changes** |
| --- | --- | --- | --- | --- |
| 1 | F | 206.7 | 232.1 | 12.3 |
| 2 | M | 284.6 | 309.9 | 8.9 |
| 3 | M | 155.5 | 300.1 | 93.0 |
| 4 | M | 322.2 | 473.3 | 46.9 |
| 5 | M | 363.6 | 411.1 | 13.1 |
| 6 | M | 282.3 | 420.6 | 49.0 |
| 7 | M | 227.8 | 317.1 | 39.2 |
| 8 | M | 350.1 | 386.1 | 10.3 |
| 9 | F | 286.1 | 366.3 | 28.0 |
| 10 | M | 291.2 | 390.5 | 34.1 |
| 11 | M | 238.4 | 450.0 | 88.8 |
| 12 | M | 207.5 | 365.0 | 75.9 |
| 13 | M | 267.6 | 291.3 | 8.9 |
| 14 | M | 243.3 | 379.3 | 55.9 |
| 15 | F | 288.0 | 329.5 | 14.4 |
| 16 | M | 277.1 | 433.3 | 56.4 |
| 17 | M | 262.3 | 306.0 | 16.6 |
| 18 | M | 131.9 | 229.5 | 74.0 |
| 19 | M | 303.8 | 323.2 | 6.4 |
| 20 | M | 264.1 | 334.6 | 26.7 |
| 21 | M | 226.2 | 436.4 | 92.9 |
| 22 | M | 168.5 | 241.9 | 43.6 |
| 23 | M | 243.9 | 378.0 | 55.0 |
| 24 | M | 239.1 | 334.4 | 39.8 |
| 25 | F | 304.3 | 399.1 | 31.2 |
| 26 | M | 270.3 | 319.3 | 18.1 |
| 27 | M | 161.9 | 323.9 | 100.1 |
| 28 | M | 200.2 | 303.5 | 51.6 |
| 29 | M | 184.1 | 318.0 | 72.8 |
| 30 | M | 126.0 | 314.8 | 149.8 |
| 31 | M | 341.7 | 352.0 | 3.0 |
| 32 | M | 309.1 | 378.3 | 22.4 |
| 33 | M | 132.4 | 264.5 | 99.8 |
| 34 | M | 306.8 | 354.2 | 15.4 |
| 35 | M | 281.9 | 315.5 | 11.9 |
| 36 | F | 305.9 | 330.2 | 8.0 |
| 37 | M | 339.8 | 346.8 | 2.1 |
| 38 | F | 265.9 | 445.7 | 67.6 |
| 39 | F | 256.5 | 324.8 | 26.6 |
| 40 | F | 266.8 | 336.1 | 26.0 |
| 41 | F | 219.2 | 242.9 | 10.8 |
| 42 | M | 219.7 | 379.8 | 72.9 |
| 43 | M | 305.2 | 406.4 | 33.1 |
| 44 | M | 310.5 | 333.7 | 7.5 |
| 45 | M | 216.5 | 343.4 | 58.6 |
| **Shapiro-Wilk test** | | 0.21 | 0.43 | 0.001 |

**Table S2.** The average elastic modulus, in kPa, of the **medial gastrocnemius** muscle before and immediately after a heel drop exercise (n=45) (raw data).

| **Subjects** | **Sex** | **Pre-** | **Post-** | **% change** |
| --- | --- | --- | --- | --- |
| 1 | F | 20.5 | 31.9 | 55.5 |
| 2 | M | 27.8 | 39.7 | 42.8 |
| 3 | M | 16.1 | 25.0 | 55.4 |
| 4 | M | 15.3 | 22.1 | 44.4 |
| 5 | M | 10.8 | 28.8 | 165.9 |
| 6 | M | 12.7 | 28.9 | 127.3 |
| 7 | M | 18.0 | 29.3 | 62.9 |
| 8 | M | 15.4 | 44.3 | 187.9 |
| 9 | F | 16.4 | 27.4 | 66.6 |
| 10 | M | 21.5 | 29.7 | 37.9 |
| 11 | M | 13.5 | 38.1 | 181.6 |
| 12 | M | 16.9 | 34.0 | 100.8 |
| 13 | M | 21.0 | 26.7 | 27.5 |
| 14 | M | 16.3 | 24.3 | 49.3 |
| 15 | F | 17.0 | 31.1 | 83.4 |
| 16 | M | 18.6 | 25.8 | 38.9 |
| 17 | M | 22.6 | 26.4 | 16.9 |
| 18 | M | 17.0 | 22.4 | 31.9 |
| 19 | M | 11.9 | 25.3 | 112.4 |
| 20 | M | 12.0 | 28.9 | 140.0 |
| 21 | M | 14.3 | 32.3 | 125.7 |
| 22 | M | 15.5 | 29.8 | 92.8 |
| 23 | M | 13.2 | 32.5 | 147.0 |
| 24 | M | 13.3 | 26.4 | 98.3 |
| 25 | F | 15.3 | 32.1 | 110.0 |
| 26 | M | 13.9 | 18.9 | 36.5 |
| 27 | M | 19.6 | 24.0 | 22.3 |
| 28 | M | 14.7 | 31.9 | 116.8 |
| 29 | M | 20.8 | 31.0 | 48.8 |
| 30 | M | 26.9 | 28.7 | 6.8 |
| 31 | M | 16.2 | 26.8 | 65.7 |
| 32 | M | 21.6 | 31.9 | 47.9 |
| 33 | M | 14.3 | 17.6 | 22.6 |
| 34 | M | 21.5 | 36.8 | 71.3 |
| 35 | M | 32.7 | 38.5 | 17.6 |
| 36 | F | 13.3 | 17.9 | 34.5 |
| 37 | M | 20.6 | 28.4 | 38.2 |
| 38 | F | 18.1 | 22.3 | 23.0 |
| 39 | F | 18.0 | 30.4 | 68.4 |
| 40 | F | 14.8 | 26.5 | 78.5 |
| 41 | F | 16.7 | 33.0 | 97.8 |
| 42 | M | 14.7 | 35.5 | 141.3 |
| 43 | M | 15.2 | 25.0 | 64.3 |
| 44 | M | 13.2 | 31.3 | 137.9 |
| 45 | M | 17.0 | 22.0 | 29.6 |
| **Shapiro-Wilk test** | | 0.02* | 0.87* | 0.01 |
| *Cases with outlier excluded from the normality assessment | | | | |

**Table S3.** The average elastic modulus, in kPa, of the **lateral gastrocnemius** muscle before and immediately after a heel drop exercise (n=45) (raw data).

| **Subjects** | **Sex** | **Pre-** | **Post-** | **% change** |
| --- | --- | --- | --- | --- |
| 1 | F | 22.5 | 24.8 | 10.1 |
| 2 | M | 15.8 | 36.2 | 129.1 |
| 3 | M | 18.8 | 24.9 | 32.4 |
| 4 | M | 14.1 | 25.8 | 83.4 |
| 5 | M | 13.0 | 24.9 | 91.8 |
| 6 | M | 11.8 | 28.7 | 142.9 |
| 7 | M | 15.1 | 21.2 | 39.9 |
| 8 | M | 16.9 | 38.4 | 126.8 |
| 9 | F | 12.9 | 30.3 | 135.7 |
| 10 | M | 18.4 | 31.4 | 71.1 |
| 11 | M | 11.5 | 26.8 | 133.0 |
| 12 | M | 15.9 | 36.0 | 125.8 |
| 13 | M | 12.2 | 19.0 | 56.4 |
| 14 | M | 13.4 | 18.9 | 41.0 |
| 15 | F | 12.8 | 34.3 | 167.1 |
| 16 | M | 19.7 | 19.9 | 1.0 |
| 17 | M | 18.4 | 22.1 | 20.5 |
| 18 | M | 23.3 | 29.4 | 26.2 |
| 19 | M | 20.4 | 21.9 | 7.7 |
| 20 | M | 19.3 | 20.4 | 5.9 |
| 21 | M | 12.4 | 24.5 | 97.8 |
| 22 | M | 16.9 | 28.6 | 69.5 |
| 23 | M | 14.0 | 32.9 | 135.5 |
| 24 | M | 15.9 | 23.5 | 47.9 |
| 25 | F | 11.7 | 29.5 | 151.9 |
| 26 | M | 13.8 | 20.3 | 47.6 |
| 27 | M | 19.6 | 19.9 | 1.5 |
| 28 | M | 21.0 | 26.9 | 27.8 |
| 29 | M | 15.5 | 28.5 | 83.9 |
| 30 | M | 16.0 | 37.1 | 131.9 |
| 31 | M | 20.2 | 29.4 | 45.4 |
| 32 | M | 10.8 | 14.7 | 35.8 |
| 33 | M | 11.0 | 14.6 | 33.0 |
| 34 | M | 21.6 | 25.1 | 16.2 |
| 35 | M | 18.4 | 25.0 | 36.1 |
| 36 | F | 11.8 | 19.1 | 62.3 |
| 37 | M | 27.2 | 37.9 | 39.3 |
| 38 | F | 17.5 | 27.2 | 55.4 |
| 39 | F | 14.0 | 25.4 | 80.8 |
| 40 | F | 9.1 | 23.8 | 161.0 |
| 41 | F | 18.7 | 20.4 | 9.1 |
| 42 | M | 18.4 | 34.8 | 89.0 |
| 43 | M | 12.1 | 27.5 | 127.0 |
| 44 | M | 10.8 | 29.5 | 173.4 |
| 45 | M | 17.7 | 21.0 | 18.5 |
| **Shapiro-Wilk test** | | 0.20 | 0.30 | 0.01 |
